# Pioglitazone Attenuates Reoxygenation Injury in Renal Tubular NRK-52E Cells Exposed to High Glucose *via* Inhibiting Oxidative Stress and Endoplasmic Reticulum Stress

**DOI:** 10.3389/fphar.2019.01607

**Published:** 2020-01-23

**Authors:** Cong Zou, Zhiyu Zhou, Yunming Tu, Weichao Wang, Tongchang Chen, Honglin Hu

**Affiliations:** ^1^Department of Endocrinology, the Fourth Affiliated Hospital of Nanchang University, Nanchang, China; ^2^Department of Pathology, College of Traditional Chinese Medicine, Jiangxi University of Traditional Chinese Medicine, Nanchang, China; ^3^Department of Urology, the Second Affiliated Hospital of Nanchang University, Nanchang, China

**Keywords:** pioglitazone, hypoxia/reoxygenation injury, high glucose, endoplasmic reticulum stress, oxidative stress

## Abstract

Renal ischemia-reperfusion injury is a major cause of acute kidney injury. In the present study, we investigated the effects of pioglitazone on hypoxia/reoxygenation (H/R) injury in rat renal tubular epithelial cells (RTECs) under normal- (NG) or high-glucose (HG) culture conditions *via* evaluating oxidative stress and endoplasmic reticulum stress (ERS). The RTECs (NRK-52E cells) were divided into six groups as follows: NG group, HG group, NG + H/R group, HG + H/R group, NG + Pio + H/R group, and HG + Pio + H/R group, among which cells in H/R groups were subjected to 4 h of hypoxia followed by 12 h of reoxygenation. After that, the cells were evaluated using the Cell Counting Kit-8 assay for the determination of their viability and flow cytometry assay for the detection of apoptosis. The levels of superoxide dismutase (SOD), glutathione reductase (GSH), catalase (CAT), and malondialdehyde (MDA) were determined *via* colorimetric chemical assays. In addition, the expression of ERS-associated proteins, i.e. ATF4, ATF6, GRP78, and CHOP, was determined *via* western blotting. A HG environment could reduce the viability and increase the apoptotic rate of NRK-52E cells with increased MDA levels and decreased SOD, CAT, and GSH levels, and upregulate the expression of ERS-associated proteins, i.e. ATF4, ATF6, and GRP78. H/R injury could further aggravate changes in the above indicators, but pioglitazone could significantly reverse such changes and alleviate cell injury. Thus, Pioglitazone exhibits a cytoprotective effect on RTECs against H/R injury under NG or HG culture conditions by inhibiting oxidative stress and ERS.

## Introduction

Diabetes is associated with numerous long-term complications, including kidney disease, cardiovascular diseases, retinopathy, and stroke; all of which reduce the quality of life and the survival rate of patients (Yazdanpanah et al., 2017). In addition, diabetes has a high tendency to cause renal ischemia-reperfusion injury (IRI) (Zhang et al., 2017). To date, there have been numerous studies reporting drugs that exhibit protective effects against renal IRI (Lima-Posada et al., 2019; Tajima et al., 2019; Wei et al., 2019), but there is still no specific drug used in clinical practice for the prevention and treatment of renal IRI. Our previous studies demonstrated that the peroxisome proliferator-activated receptor gamma (PPAR-γ) activator pioglitazone can protect normal mice against renal IRI *via* its anti-apoptotic and antioxidant activities (Hu et al., 2012; Zou et al., 2013). Tawfik reported that pioglitazone alleviates acute IRI-induced renal injuries in diabetic rats *via* the modulation of oxidative stress and inflammation (Tawfik, 2012).

Hypoxia/reoxygenation (H/R) injury in rat renal tubular epithelial cells (RTECs) is an important pathological event in renal IRI, involving apoptosis, excessive production of reactive oxygen species (ROS), and endoplasmic reticulum stress (ERS) (Xu et al., 2017). It has been previously shown that the activation of PPAR-γ by pioglitazone can alleviate ERS in the islet cells of diabetic mice (Evans-Molina et al., 2009). The activation of PPAR-γ by pioglitazone exhibits protective effects against gastric mucosal IRI by inducing ERS (Naito et al., 2011). Therefore, our study aimed to investigate the effects of pioglitazone on H/R injury in RTECs (NRK-52E cells) under normal- (NG) or high-glucose (HG) culture conditions *via* evaluating oxidative stress and ERS.

## Materials and Methods

### Reagents

NRK-52E cells were purchased from the Cell Bank of Shanghai Institutes for Biological Sciences, Chinese Academy of Sciences. Pioglitazone solution (purity 99%) was purchased from Jiangsu Hengrui Medicine Co., Ltd. (China). Rabbit anti-mouse ATF4, ATF6, GRP78, and CHOP antibodies, as well as β-actin antibody were purchased from Abcam plc. (USA). Superoxide dismutase (SOD), catalase (CAT), glutathione reductase (GSH), and malondialdehyde (MDA) assay kits were purchased from the Nanjing Jiancheng Bioengineering Institute (China).

### Cell Culture

NRK-52E cells were cultured in Dulbecco's modified Eagle's medium (DMEM) containing 1 g/L of D-glucose and 10% newborn calf serum (NBCS) or HG DMEM (containing 4.5 g/L of D-glucose) in 50-mL culture flasks at 37°C in a CO_2_ (5%, v/v) incubator.

### Experimental Groups

RTECs (NRK-52E cells) (four to six passages passages) grown to the logarithmic phase were randomly divided into the following six groups: 1. NG group: Cells cultured in NG medium (containing 1 g/L of D-glucose); 2. HG group: Cells cultured in HG medium (containing 4.5 g/L of D-glucose); 3. NG + H/R group: Cells cultured in NG medium and subjected to H/R treatment (in accordance with our previous pre-experimental results, i.e. 4 h of hypoxia followed by 12 h of reoxygenation); 4. HG + H/R group: Cells cultured in HG medium for 48 h followed by H/R treatment; 5. NG + Pio + H/R group: Cells cultured in NG medium followed by the addition of pioglitazone solution (Hengrui Medicine Co., Ltd., China) [to a final concentration of 40 mmol/L, which was selected based on our previous study (Xi et al., 2019)] 1 h prior to H/R treatment; 6. HG + Pio + H/R group: Cells cultured in HG medium followed by the addition of pioglitazone solution (to a final concentration of 40 mmol/L) 1 h prior to H/R treatment. All experiments were repeated five times (n = 5).

### Construction of H/R Model

D-Hanks solution was employed for preparing the hypoxic solution. Briefly, the D-Hanks solution bubbled with N_2_ to remove the O_2_ was equilibrated in a closed container for more than 1 h into a hypoxic solution. After removing the oxygen-containing medium *via* aspiration, the hypoxic solution was added into the culture flask, which was then hypoxically incubated at 37°C for 4 h in an anaerobic chamber equilibrated with 5% CO_2_ and 95% N_2_. A metal catalyzer (Engelhard, USA) was used to maintain a constant low oxygen concentration (< 0.1%) in the chamber. Subsequently, the hypoxic solution was replaced with normal or HG medium and the culture flasks were returned to the incubator for 12 h of re-oxygenation.

### Cell Viability Assay

The Cell Counting Kit-8 (CCK-8) assay was carried out in accordance with the manufacturer's instructions. Briefly, the cells were inoculated into a 96-well plate and 10 μL of CCK-8 reagent was added to each well, followed by incubation at 37°C for 4 h prior to the measurement of absorbance at a wavelength of 450 nm (A_450_).

### Flow Cytometric Detection of Apoptosis

The harvested cells were washed twice with 1 mL of phosphate-buffered saline (PBS) each time and centrifuged at 1,500 rpm for 3 min. The resulting cell pellet was re-suspended in 300 µL of pre-chilled 1× binding buffer, and each tube of the cell suspension was mixed gently with 3 µL of Annexin V-FITC and 5 µL of propidium iodide-phycoerythrin (PI-PE), followed by incubation in the dark at room temperature for 10 min. Each tube was subsequently mixed evenly with 200 µL of pre-chilled 1× binding buffer prior to being loaded onto the flow cytometer.

### Determination of Intracellular Levels of Antioxidant Enzymes and MDA in Each Group

The harvested cells were lysed in a lysis buffer and centrifuged to obtain the protein samples, which were then quantified *via* the Coomassie Brilliant Blue assay (Bradford assay). After that, the intracellular levels of SOD, GSH, CAT, and MDA were determined *via* colorimetric chemical assays according to the manufacturer's instructions.

### Determination of the Expression of ERS-Associated Proteins *via* Western Blotting

The harvested cells were lysed in a lysis buffer and centrifuged to obtain the protein samples, which were then quantified *via* the Coomassie Brilliant Blue assay (Bradford assay). The protein samples were loaded at 200 μg per well and separated *via* electrophoresis in a 10–15% sodium dodecyl sulfate-polyacrylamide gel (SDS-PAGE). Subsequently, the proteins were transferred onto a nitrocellulose membrane, which was then blocked in Tris Buffered Saline with Tween 20 (TBST) buffer containing 5% skimmed milk powder and incubated overnight at 4°C with the primary antibody. Finally, the membrane was incubated with horseradish peroxidase-conjugated secondary antibody (diluted to 1:1,000) and imaged using the LAS-3000 Chemiluminescence Image Analyzer for western blotting (Fujifilm Holdings Corporation, Japan). The protein bands were quantitatively analyzed using Fujifilm Multi-Gauge software with β-actin serving as the internal control.

### Statistical Analyses

Statistical analyses in this study were performed using GraphPad Prism 5.0 statistical software. All statistical data were expressed as means ± SE. The comparison between groups was carried out using one-way analysis of variance (ANOVA) plus the Tukey post-hoc multiple-comparisons test. *P* < 0.05 indicates the presence of statistically significant differences.

## Results

### Pioglitazone Improved the Viability of H/R-Treated Cells

**Figure 1:**
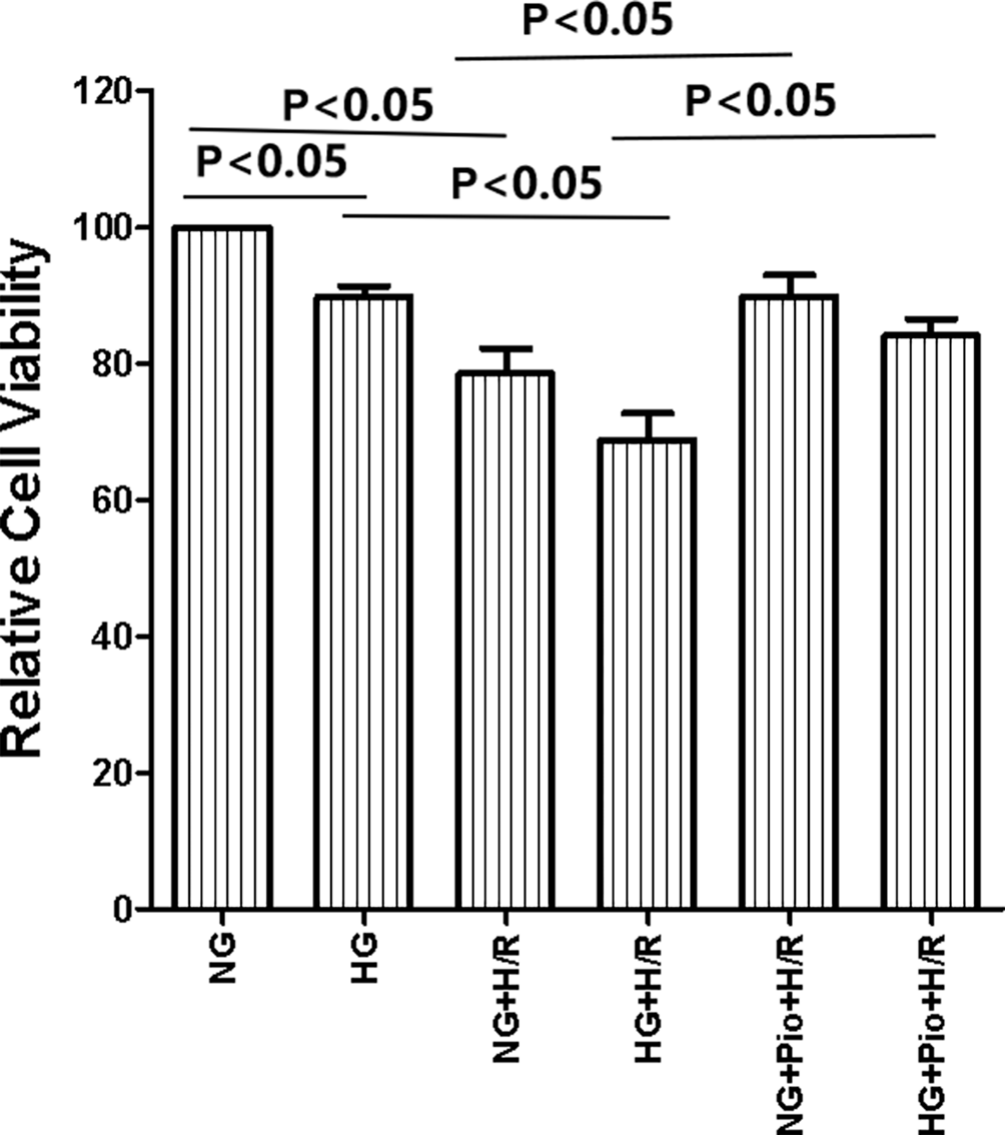
Pioglitazone improved the viability of cells with H/R injury. Values are expressed as means ± SE (n = 5).

The results of the CCK-8 assay showed that the HG group had lower cell viability than the NG group, and there was a statistically significant difference between the two groups. After being subjected to H/R treatment, the viability of cells in both NG and HG groups showed statistically significant declines. However, the viability of H/R-treated cells was significantly improved by pioglitazone treatment (**Figure 1**).

### Pioglitazone Reduced the Apoptotic Rate of H/R-Treated Cells

**Figure 2:**
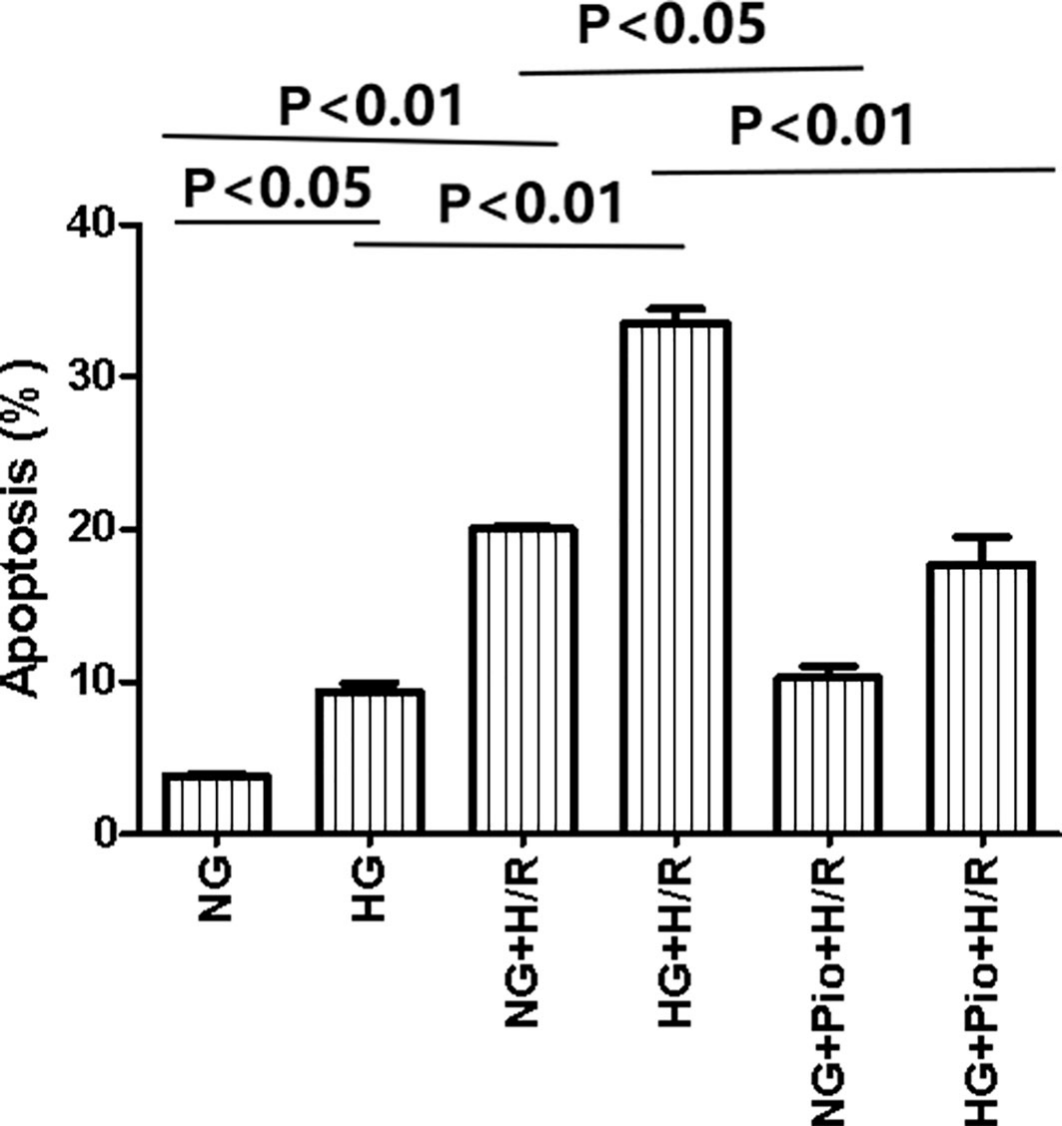
Pioglitazone reduced apoptosis in cells with H/R injury. Values are expressed as means ± SE (n = 5).

The apoptosis of cells in each group was detected *via* flow cytometry assay, and the results showed that the HG group cells had a statistically significantly higher apoptotic rate than the NG group cells. Both NG and HG groups showed statistically significant increases in the apoptotic rate following H/R treatment, but the apoptotic rate of H/R-treated cells was significantly reduced by the pioglitazone treatment (**Figure 2**).

### Pioglitazone Increased the SOD, CAT, and GSH Levels, and Reduced the MDA Level in H/R-Treated Cells

**Figure 3:**
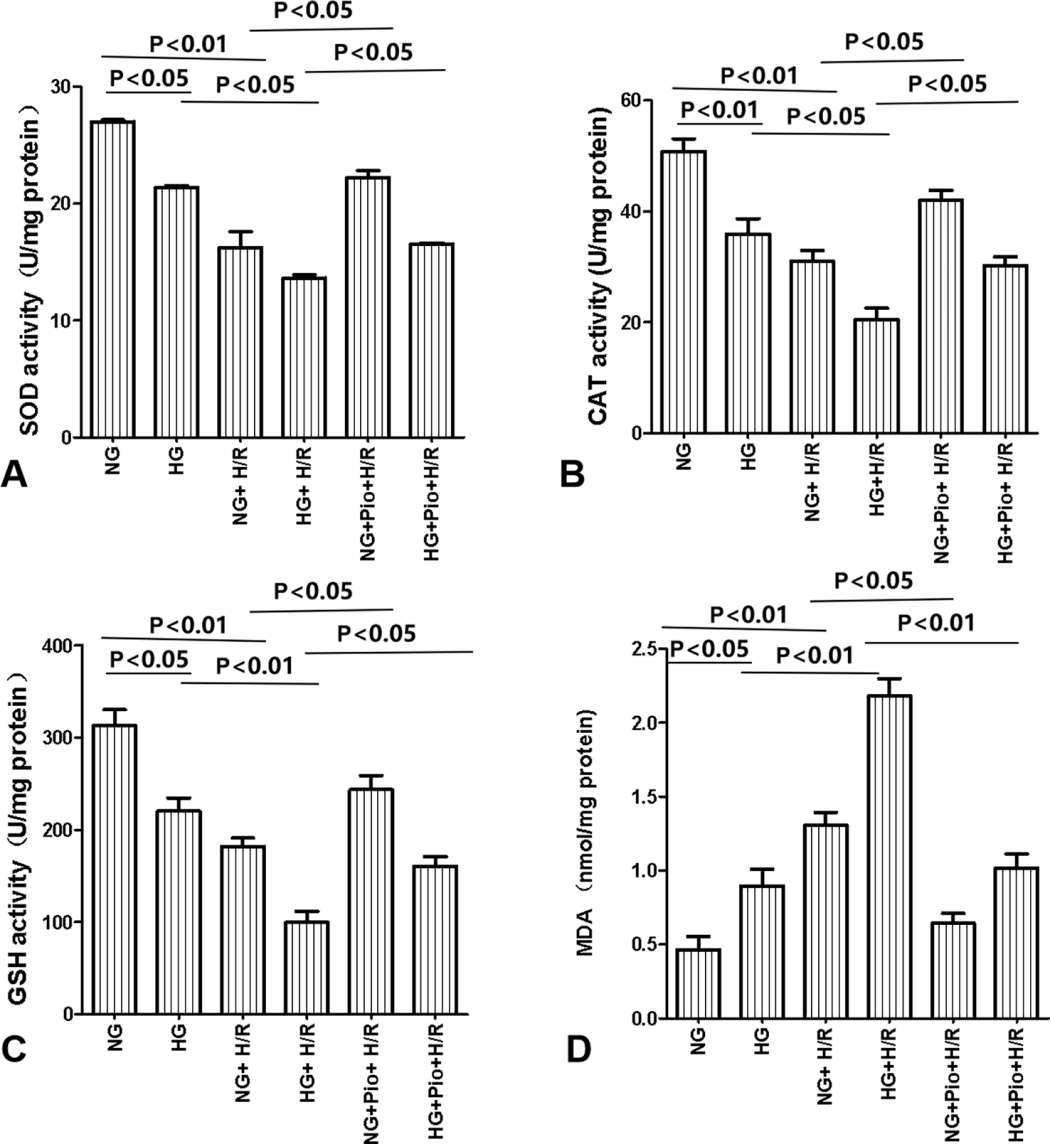
Pioglitazone increased the levels of SOD **(A)**, CAT **(B)**, and GSH **(C)** and reduced the level of MDA **(D)** in cells with H/R injury. Values are expressed as means ± SE **(A**–**D)** (n = 5).

The intracellular levels of SOD, CAT, GSH, and MDA were determined *via* colorimetric chemical assays. The HG group cells displayed lower levels of SOD, CAT, and GSH, as well as a higher level of MDA than the NG group cells, with statistically significant differences. Following H/R treatment, both NG and HG groups showed further declines in intracellular SOD, CAT, and GSH levels, and a further increase in the intracellular level of MDA, with statistically significant differences. However, the pioglitazone treatment significantly increased the levels of SOD, CAT, and GSH, and reduced the level of MDA in H/R-treated cells (**Figure 3**).

### Pioglitazone Reduced the Expression of ATF4, ATF6, and GRP78 Proteins in H/R-Treated Cells

**Figure 4:**
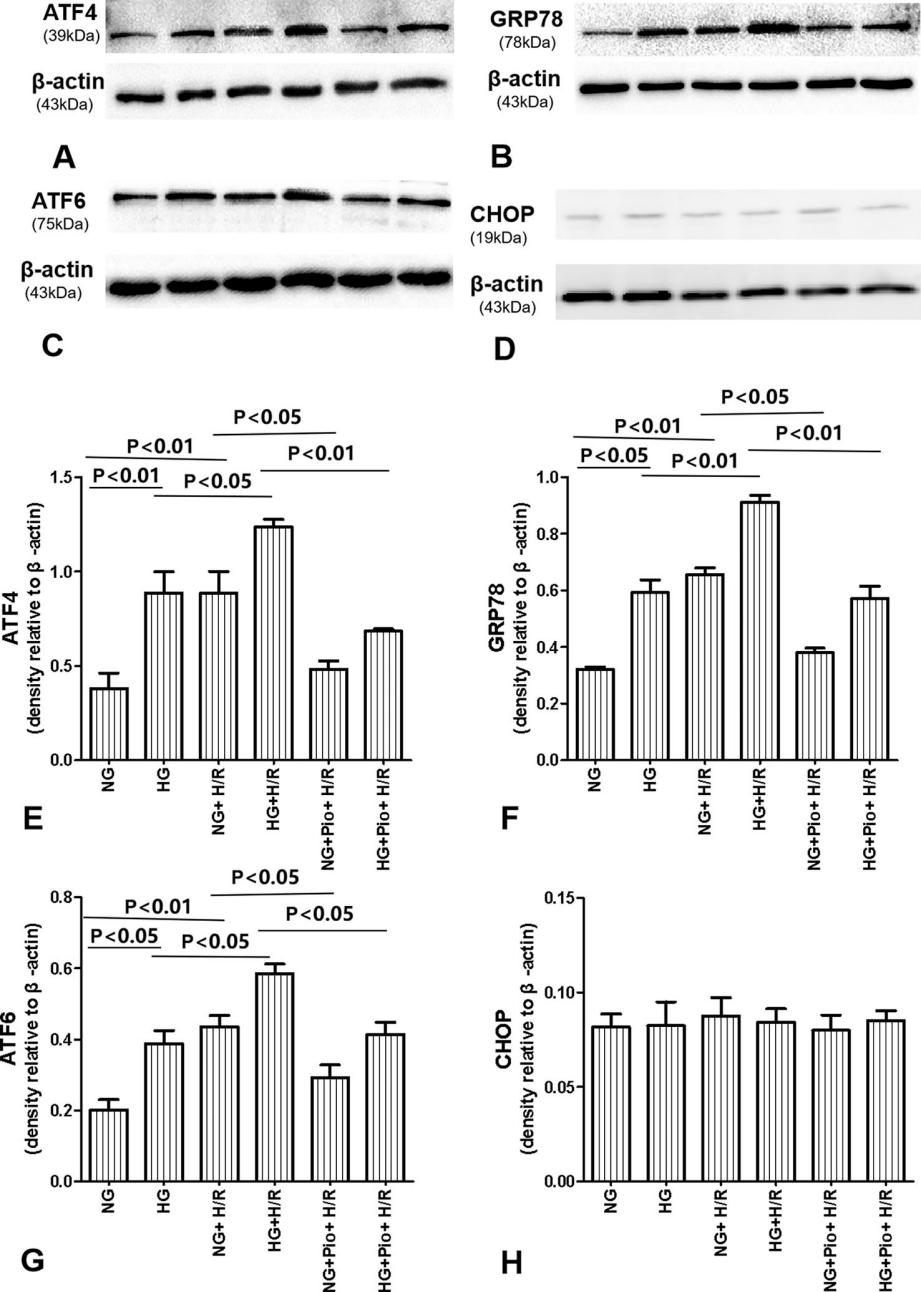
Pioglitazone reduced the expression of ATF4, ATF6, and GRP78 proteins in cells with H/R injury, but there was no significant difference in the expression of CHOP protein between groups. **(A**–**D)** Representative western blots show ATF4, ATF6, GRP78, and CHOP protein levels. **(E**–**H)** Quantitative analysis of ATF4, ATF6, GRP78, and CHOP protein levels. Values are expressed as means ± SE **(E**–**H)** (n = 5).

The expression of ERS-related proteins, i.e. ATF4, ATF6, GRP78, and CHOP, was determined *via* western blotting. The HG group cells displayed statistically significantly higher expression levels of ATF4, ATF6, and GRP78 proteins than the NG group cells. The expression levels of ATF4, ATF6, and GRP78 proteins in both NG and HG groups were further elevated following the H/R treatment, with statistically significant differences. However, the pioglitazone treatment significantly reduced the expression of ATF4, ATF6, and GRP78 proteins in H/R-treated cells. On the other hand, there was no significant difference in the expression of CHOP protein between groups (**Figure 4**).

## Discussion

Our previous studies showed that the PPAR-γ agonist pioglitazone could protect normal mice against renal IRI by inhibiting apoptosis and improving antioxidant effects (Hu et al., 2012; Zou et al., 2013). In this study, we further clarified that pioglitazone could alleviate H/R injury in RTECs under NG and HG culture conditions by inhibiting the ERS and enhancing the antioxidant activity.

Pioglitazone is a thiazolidinedione anti-diabetic drug that has been shown to reduce blood glucose and inflammation markers in animal models and in diabetic kidney transplant patients *via* its anti-inflammatory and anti-fibrotic effects (Kharazmkia et al., 2014; Sun et al., 2017). A low level of pioglitazone is sufficient to activate and facilitate the binding of PPAR-γ to its receptors, and to recruit and regulate the transcriptional activities of synergistic factors. It has been shown that pioglitazone could improve diabetic kidney diseases by inhibiting insulin resistance or oxidative stress (Ali et al., 2016). Previous studies also showed that pioglitazone exhibits a protective effect against IRI *via* its anti-apoptotic, anti-inflammatory, and antioxidant activities (Reel et al., 2013; Ali et al., 2018; Pane et al., 2018). In this study, we created a model of renal IRI by cultivating RTECs with H/R injury under HG conditions. The results showed that pioglitazone-pretreated cells had significantly elevated activities of antioxidant enzymes following H/R treatment. Additionally, the elevation of MDA (MDA is the end-product of lipid peroxidation and is widely applied for determining the effects of oxidative stress on the mitochondrial respiratory chain) in pioglitazone-pretreated cells was suppressed following H/R treatment.

Renal IRI may result in energy depletion, disorders of intracellular calcium homeostasis, and accumulation of free radicals, thereby triggering the onset of ERS (Yan et al., 2018). In the early stage of injury, the ERS marker protein GRP78 dissociates from the transmembrane receptor of the endoplasmic reticulum and binds to unfolded/misfolded proteins to promote the degradation of misfolded proteins and trigger the unfolded protein response (UPR), thereby alleviating the intracellular stress (Walter and Ron, 2011). In the event that the cellular functions cannot be restored, the UPR activates the pro-apoptotic pathway that ultimately leads to apoptosis (Avila et al., 2013: Ye et al., 2013). Moreover, in response to ERS, the UPR, which can minimize the accumulation and aggregation of misfolded proteins, is activated. The UPR consists of three highly coordinated signaling pathways including PERK, IRE1α, and ATF6 pathways. These three key proteins are also responsible for the proapoptotic pathways of UPR during ERS-mediated apoptosis caused by prolonged stress (Bernales et al., 2006). In the present study, we detected the expression of ATF4, ATF6, GRP78, and CHOP by Western blot, and as a result, ATF4, ATF6, and GRP78 proteins were all significantly increased after H/R injury. H/R injury also increased cell apoptosis and decreased cell viability compared to control group. However, pioglitazone treatment suppressed the expressions of ATF4, ATF6, and GRP78 and also reduced cell apoptosis, and improved cell viability compared to control group, indicating that pioglitazone exerted protective effects against H/R injury in RTECs through suppressing ERS-induced apoptosis.

Hyperglycemia is an independent risk factor leading to acute kidney injury (AKI) (James et al., 2015). Previous animal experimentation showed that diabetes aggravates cerebral IRI in rats, probably by enhancing the ERS (Srinivasan and Sharma, 2011). In this study, we found that the HG + H/R group had significantly lower viability of RTECs, with significantly higher apoptosis and ERS than that in the NG + H/R group. Hence, it was observed that H/R injury in RTECs was further aggravated under the HG environment. Pioglitazone could significantly downregulate the expression of ERS markers, i.e. ATF4, ATF6, and GRP78 proteins, and reduce cell apoptosis with H/R injury under HG conditions. However, we found in this study that the ERS-specific protein CHOP displayed a low expression level, which did not differ significantly between groups. Hence, its mechanism of action still requires further elucidation. The full role of ERS is also not known, as the study by Naito (Naito et al., 2011) suggested that pioglitazone could also provide protective effects by inducing ERS rather than by reducing it.

In conclusion, The HG culture conditions aggravated the H/R injury in RTECs, and its underlying mechanism may be associated with oxidative stress and ERS. Pioglitazone can alleviate H/R injury in RTECs under NG and HG conditions by inhibiting oxidative stress and ERS.

## Data Availability Statement

The datasets generated for this study are available on request to the corresponding author.

## Author Contributions

The authors contributed in the following way: CZ, ZZ, YT, WW, TC and HH: acquisition, analysis, and interpretation of data, and drafting the paper. CZ and HH: study conception and design, analysis and interpretation of data, and drafting the paper.

## Funding

This study was funded by grants from the National Natural Science Foundation of China (No. 81560311). The funders had no role in study design, data collection and analysis, decision to publish, or preparation of the manuscript.

## Conflict of Interest

The authors declare that the research was conducted in the absence of any commercial or financial relationships that could be construed as a potential conflict of interest.
